# Applying Tai Chi as a rehabilitation program for stroke patients in the recovery phase: study protocol for a randomized controlled trial

**DOI:** 10.1186/1745-6215-15-484

**Published:** 2014-12-11

**Authors:** Yong Zhang, Hongwei Liu, Li Zhou, Kai Chen, He Jin, Yihuai Zou, Zongheng Li

**Affiliations:** Department of Rehabilitation, Dongzhimen Hospital affiliated to Beijing University of Chinese Medicine, No. 5, Haiyuncang, Dongcheng District, Beijing, 100700 China; Department of Neurology and Stroke Center, Dongzhimen Hospital affiliated to Beijing University of Chinese Medicine, No. 5, Haiyuncang, Dongcheng District, Beijing, 100700 China

**Keywords:** Recovery phase, Rehabilitation, Stroke, Tai Chi

## Abstract

**Background:**

As the second commonest cause of death and a major cause of disability worldwide, stroke has greatly influenced patients’ quality of life and created a huge public health burden. As a special form of physical activity that has been widely practiced in China, and even throughout the world, Tai Chi may be favorable for the rehabilitation of stroke patients. Several studies have been conducted to investigate the rehabilitative effects of Tai Chi for stroke patients, but none of them have been focused on the recovery phase (2 to 24 weeks) of stroke.

**Methods/design:**

This study is an assessor-blinded randomized controlled trial. A total of 50 eligible participants will be randomly assigned to either a control group or a Tai Chi group. Patients in the control group will receive standard, conventional rehabilitation therapies, and a combination of Tai Chi and conventional rehabilitation programs will be applied in the Tai Chi group. The recovery of motor impairment, functional activity and balance abilities as measured with the Fugl-Meyer Assessment, Barthel Index and Berg Balance Scale will be assessed as primary outcome measures. The secondary outcome measures to be used are the scores on the Stroke-Specific Quality of Life Scale, the National Institutes of Health Stroke Scale and the objective parameters of the RSscan footscan gait system. All assessments will be conducted at baseline, 4 weeks after the rehabilitation course and at the end of 3-month follow-up.

**Discussion:**

The results of this study will provide preliminary evidence regarding the efficacy and feasibility of Tai Chi as an additional rehabilitative program for stroke patients in the recovery phase.

**Trial registration:**

Chinese Clinical Trial Register ID: ChiCTR-TRC-13003661 (7 October 2013)

**Electronic supplementary material:**

The online version of this article (doi:10.1186/1745-6215-15-484) contains supplementary material, which is available to authorized users.

## Background

Stroke has become the second commonest cause of death and a major cause of disability worldwide[1]. It has greatly influenced stroke patients’ quality of life and created a huge public health burden[2]. With the population aging and lifestyles changing, the burden will increase greatly in the next 20 years, especially in the developing countries[3]. Recent epidemiological studies have shown that there are over 7 million stroke survivors living in China, and about 70% of them have functional disabilities[4]. This reality drove us to search for effective modalities of treatment for stroke rehabilitation.

Tai Chi (also called Taiji or Tai Chi Chuan) is a famous intangible cultural heritage that originated in China as a martial art hundreds of years ago[5]. Built upon the mind–body connection, Tai Chi combines physical movement, meditation and breathing to induce relaxation and tranquility of the mind, and it improves balance, postural control, movement coordination, strength and flexibility[6]. In the past decade, a substantial number of studies and reviews have been conducted in the field of the clinical use of Tai Chi[7–11]. Recently, the significant effects of Tai Chi for fibromyalgia and Parkinson’s disease rehabilitation have been confirmed, and related studies published in the *New England Journal of Medicine* have brought great attention to and general agreement on the clinical effects of Tai Chi[12, 13].

Physical activity is an important component of comprehensive stroke rehabilitation programs implemented to reduce disabilities. As a special form of physical activity that has been widely practiced in China, and even throughout the world, Tai Chi may be favorable for the rehabilitation of stroke patients[9, 14, 15]. Tai Chi is considered a complex, multicomponent intervention that integrates physical, psychosocial, emotional, spiritual and behavioral elements[12]. The main essence of Tai Chi practice is similar to Bobath therapy and proprioceptive neuromuscular facilitation techniques, which made it possible for us to apply Tai Chi for stroke rehabilitation[9].

Several studies have been conducted to explore the health-enhancing qualities of Tai Chi for stroke patients. The studies conducted by Taylor *et al*. demonstrated that community-based Tai Chi practice was a safe and feasible program for stroke patients[16, 17]. Another study indicated that a 12-posture short-form Tai Chi exercise was helpful in enhancing the standing balance of patients with a stroke history longer than 6 months after onset[18]. To the best of our knowledge, there has been no study to date in which Tai Chi was used in a rehabilitation program for stroke patients in the recovery phase (2 to 24 weeks). In this study protocol article, we will describe the rationale, design and analytic methods of a randomized controlled trial designed to investigate the rehabilitative effects of Tai Chi among hospitalized stroke patients in the recovery phase. We hypothesized that patients in the Tai Chi group would demonstrate better improvement compared with that in the control group.

## Methods/design

### Study design and setting

**Figure 1:**
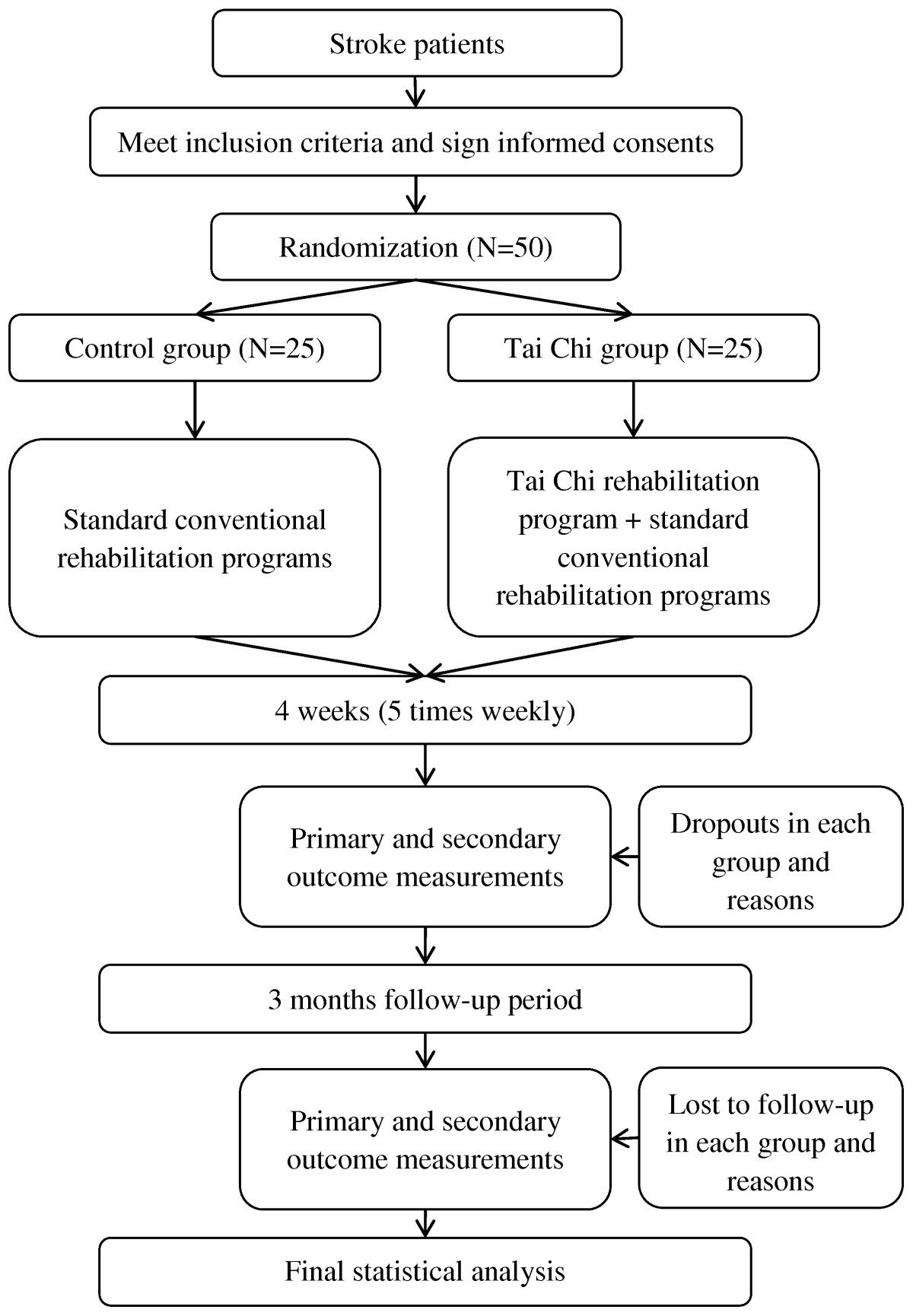
**Flowchart of the study design.**

The study will be conducted from 1 January 2014 to 31 December 2014 in Dongzhimen Hospital, which is affiliated with the Beijing University of Chinese Medicine. All participants will be allocated in a 1:1 ratio into either a control group or a Tai Chi group. Patients in the control group will receive standard conventional rehabilitation therapies, and a combination of Tai Chi and conventional rehabilitation therapies will be applied in the Tai Chi group. The participants’ scores on the Fugl-Meyer Assessment, Barthel Index and Berg Balance Scale will be assessed as primary outcome measures. The secondary outcome measures will be scores on the Stroke-Specific Quality of Life Scale, the National Institutes of Health Stroke Scale and the objective parameters of the RSscan footscan gait system (RSscan International, Olen, Belgium). All assessments will be conducted at baseline, 4 weeks after the rehabilitation course and at the end of 3-month follow-up. The study design is summarized in Figure 1.

### Ethics

The study protocol was approved by the research ethics committee of Dongzhimen Hospital, which is affiliated with the Beijing University of Chinese Medicine (no. 2013JS201), and it follows the principles of the CONSORT statements as well as the Declaration of Helsinki. The trial is registered with the Chinese Clinical Trial Register (ChiCTR-TRC-13003661). The research ethics committee will also be in charge of supervising all procedures carried out in our study, including patient recruitment, randomization, conduct of the study and data storage. In cases of changes to our study protocol, we have to hand in a written application to the research ethics committee. The committee members will then decide whether it is necessary to change the study protocol.

### Randomization and allocation concealment

This study is designed as a pilot study in which 20 participants will be included in each group. Allowing for a 25% dropout rate, we will recruit 25 patients in each group which means a total of 50 patients should be included during the whole trial[19, 20]. Participants will be assigned in a 1:1 ratio according to a computer-generated randomization list. Assignments will be sealed in opaque envelopes and will be opened by the study staff following informed consent procedures and baseline testing. The participants and researchers will know the allocated group, but the outcome assessors and data analysts will not.

### Informed consent

Prior to the study, the general study process and the responsibilities of both participants and researchers will be explained to potential participants. They will be told that their entry into the trial is entirely voluntary and that they can withdraw at any time. In the event of their withdrawal, the data collected cannot be erased and will be used in the final analyses. Written informed consent should be obtained from each participant before any interventions related to the study are started. ZL will be responsible for obtaining informed consent from all participants.

### Inclusion criteria

Participants meeting the following criteria will be included:

1. Stroke that has been confirmed by computed tomography or magnetic resonance imaging
2. Between 40 and 75 years old
3. First episode of stroke or a history of stroke, but no serious deformity, and a modified Rankin Scale grade ≤2
4. Between 2 and 20 weeks after the onset of the current stroke
5. Unilateral lower-extremity hemiplegia and able to walk ≥6 meters [18]
6. Blood pressure <160/100 mmHg
7. Sufficient cognition to follow commands, Mini Mental State Examination score >24
8. No experience with Tai Chi
9. Voluntary participation in the trial and provision of informed consent

### Exclusion criteria

Participants meeting the following criteria will be excluded:

1. Received thrombolytic therapy or surgery
2. Vital signs unstable or worsening condition
3. Severe primary diseases of the cardiovascular system, liver, kidney or hematopoietic system
4. Pregnant or lactating women
5. Participation in other clinical trials

### Interventions

#### Control group

Patients who are assigned to the control group will be engaged in standard conventional rehabilitation programs that consist of individualized amounts of various therapy options for stroke patients (for example, Bobath therapy or proprioceptive neuromuscular facilitation) provided by occupational and physical therapists. The conventional rehabilitation program consists of a series of standard rehabilitation therapies for every stroke symptom. We will select certain therapies for each of the patients according to their symptoms. The standard rehabilitation program will be carried out five times per week (that is, Monday to Friday) for 4 weeks, and each time the rehabilitation course will last for approximately 1 hour. All conventional rehabilitation programs will be conducted by two qualified therapists (YZ and HJ).

#### Tai Chi group

To study the additional effects of Tai Chi to the standard conventional rehabilitation programs, patients allocated to the intervention group will be engaged in Tai Chi rehabilitation programs in addition to the control therapies.

The Tai Chi rehabilitation program is specially established for stroke patients with lower-extremity hemiplegia by an expert panel comprising a qualified Tai Chi instructor, an experienced therapist from the Department of Rehabilitation at Dongzhimen Hospital and a senior doctor from the hospital’s stroke center. The Tai Chi rehabilitation program consists of six Tai Chi movements listed in order of difficulty. The six Tai Chi movements are Commencing, Part the Wild Horse’s Mane, Wave Hands Like Clouds, Brush Knee and Twist Step, Reverse Reeling Forearm and Closing derived from the 24 forms of simplified Tai Chi recommended as a popular health sport by the General Administration of Sport of China[7].

Our goal of selecting such movements to design a Tai Chi rehabilitation program is to help stroke patients with lower-extremity hemiplegia to maintain motor function and postural balance control. Thus, in the actual practice of the Tai Chi rehabilitation program, we will not focus on the continuity between different movements, but will put much emphasis on the accuracy and quality of performing every single movement. Each movement will be integrated therapeutically, especially for the stroke-affected side, by performing symmetrical and coordinated movements such as trunk rotation and weight-bearing, controlled and coordinated displacement of the body’s center of mass, and rebuilding of the affected ankle and knee joints by anteroposterior and mediolateral stepping[13].

The Tai Chi rehabilitation program will take place five times per week (from Monday to Friday) for 4 weeks, and each session will include a warm-up for 10 minutes, specified Tai Chi exercises performed for 40 minutes and cooling down for 10 minutes. The Tai Chi rehabilitation program will be applied 20 to 30 minutes after the patients finish the conventional rehabilitation programs. An experienced Tai Chi instructor who has been qualified and engaged in the teaching of Tai Chi courses for 10 years will lead the Tai Chi rehabilitation program. In the first week of the program, we will emphasize primarily learning and practicing single forms of Tai Chi exercises with multiple repetitions. Instruction will cover learning new forms and reviewing and practicing forms learned in previous sessions. For the remaining 3 weeks, the focus will be on performing individual forms to enhance motor function recovery and postural balance. Natural breathing will also be emphasized as part of the Tai Chi rehabilitation program. Patients will be told to breathe as they usually do so that they can focus more on the Tai Chi movements and achieve better functional recovery.

### Follow-up

After the 4-week supervised rehabilitation courses in the hospital, all patients will be discharged from the hospital and begin an additional 3-month unsupervised follow-up period. Owing to the specificity of stroke, patients are always encouraged to attend community-based rehabilitation courses. For patients in both of our study groups, we will encourage them to attend such standard rehabilitation courses. Meanwhile, patients in the Tai Chi group will be encouraged to maintain their Tai Chi practice, and they will be asked to fill out forms to record the times and durations of their Tai Chi practice. Patients in the control group will be asked to fill out similar forms to record their rehabilitation program attendance. All forms will be returned to the researchers for monitoring at the end of the follow-up period.

### Outcome measures

Data collection will be performed by a trained, certified assessor who will be blinded to patients’ group assignment at baseline, after the intervention (4 weeks) and at the end of follow-up (3 months).

### Basic characteristics variables

Demographic information of the participants will be collected at baseline to describe the sample, compare conditions and investigate characteristics associated with outcomes. These measures will include participants’ sex, age, ethnicity, marital status, educational background, time since the attack of stroke, use of medication and other details. Vital signs such as respiration rate, pulse rate, resting blood pressure and body temperature will be measured by nurses.

### Primary outcomes

The first primary outcome measurement is the recovery of motor impairment. We will apply the Fugl-Meyer Assessment (FMA) to measure it. Developed as the first quantitative evaluative instrument for measuring sensorimotor stroke recovery, the FMA is a well-designed, feasible and efficient clinical examination method that has been tested widely in the stroke population across different stroke recovery time points[21]. The FMA motor scale is recommended highly as a clinical and research tool for evaluating changes in motor impairment following stroke[22]. Its primary value is the 100-point motor domain, which has received the most extensive evaluation. It includes an assessment of the upper extremity and the lower extremity. In the present study, we will focus on the measurement of the lower extremity, which includes two subsections—the leg and coordination—with a total possible score of 34.

The improvements in activities of daily living that can be measured with the Barthel Index (BI) will be listed as another primary outcome measurement. The BI is a frequently used functional outcome measure of activities of daily living in stroke trials[23]. It is also widely used in clinical practice to assess baseline abilities, to quantify functional changes after rehabilitation and to inform discharge planning. The scale describes ten task items and is scored according to the amount of time or assistance required by the patient. These ten items are feeding, bathing, grooming, dressing, continence of bowels and bladder, transferring to and from a toilet, moving from wheelchair to bed and return, walking on a level surface for 45 meters and going up and down stairs. The total score ranges from 0 to 100, with lower scores representing greater nursing dependency[24].

The static and dynamic balance abilities will also be applied as primary outcome measurements. The Berg Balance Scale (BBS) is a clinical test widely used to quantitatively assess the static and dynamic balance abilities of older adults[25]. It comprises a set of 14 simple balance-related tasks, ranging from standing up from a sitting position to standing on one foot. The total score ranges from 0 to 56, with 0 to 20 corresponding to a high fall risk, 21 to 40 to a medium fall risk and 41 to 56 to a low fall risk. The BBS has been widely used to assess balance abilities in recent studies of the clinical effects of Tai Chi in therapy for various diseases, including stroke[18, 26]. It is identified as the most commonly used assessment tool across the continuum from acute care to community-based rehabilitation in stroke. The authors of a recent systematic review suggested that the BBS provides an effective and appropriate assessment of balance in patients with stroke and should be considered for use in measuring outcomes of various stroke rehabilitation interventions[27].

### Secondary outcomes

To the best of our knowledge, only scale evaluations have been used to measure the outcomes of stroke rehabilitation in most previous studies. Objective outcome measurements have been used in few of them. In the present study, we will try to measure several objective parameters by using the RSscan footscan gait system (2096 mm × 469 mm × 18 mm, 16,384 sensors, 480 Hz, RSscan International, Olen, Belgium). Consisting of a sensor array, a data collector, a walkway and a computer, the footscan gait system is sensitive in detecting the parameters of plantar pressure data and time phase when subjects are standing or walking on the plate. It is designed to test the characteristics of different kinds of movement, especially among athletes[28]. Nowadays, it is also used in clinical studies to provide quantitative assessments[29, 30]. In the present study, patients will be asked to stand and walk on the plate to become familiar with the system before formal data are collected. Data of plantar force distribution, impulse, maximum force, maximum pressure and time of walk phase will be collected to evaluate lower-extremity function.

We will also assess the quality of life in both groups as secondary outcomes. The Stroke-Specific Quality of Life Scale (SS-QOL) is a patient-reported outcome measure intended to provide an assessment of health-related quality of life specific to patients with stroke[31]. The SS-QOL questionnaire consists of 49 items in the 12 domains of energy, family roles, language, mobility, mood, personality, self-care, social roles, thinking, upper-extremity function, vision and work/productivity. Scoring of the SS-QOL is rated on a 5-point Likert scale. The domains are scored separately, and a total score is also calculated, with higher scores indicating better function.

The National Institutes of Health Stroke Scale (NIHSS) is a widely used, validated tool used in nearly all large clinical stroke trials to document baseline and outcome severity of neurological impairment[32, 33]. The NIHSS comprises tests of 11 items, including the levels of consciousness, selected cranial nerves, motor function, sensory function, cerebellar function, language and inattention (that is, neglect). Total scores range from 0 to 42, with scores >25 indicating very severe neurological impairment, scores of 5 to 24 suggesting moderately severe to severe impairment and scores <5 indicating mild impairment.

### Adverse events

All unexpected adverse events during the intervention period will be reported, and the causality related to the Tai Chi intervention will be analyzed. If any adverse event occurs, the Tai Chi instructor or study managers will provide the corresponding treatment to the participant. The adverse events will be immediately reported to the primary investigator and ethics committee to decide if the participant needs to withdraw from the trial. In case of stroke recurrence or other worsening conditions, patients will be stopped from participating in the study and will be referred for further treatment.

### Statistical analysis

Statistical analysis will be conducted by statisticians who are independent from the research team using SPSS 12.0 software for Windows (IBM SPSS, Chicago, IL, USA). The categorical variables will be presented with frequencies or percentages, and the continuous variables will be presented as means and standard deviations. Demographic and clinical assessments of two groups will be compared upon admission to address the baseline characteristics. For both groups, data of primary and secondary outcome measurements collected after 4 weeks and at the end of 3 months will be compared with baseline data, respectively, to evaluate the rehabilitative effects within groups. To evaluate the effects of Tai Chi on stroke rehabilitation, data of all outcome measurements after 4 weeks and at the end of 3 months will be compared between the two groups. Unpaired two-sample *t*-tests (continuous data) and χ^2^ analysis (categorical data) will be applied in data analyses between two groups. Nonparametric methods will be used when assumptions of normality are violated. Patients with missing data before 4 weeks will be eliminated. We will also conduct an intention-to-treat analysis if participants are lost to follow-up at 3 months. The statistical significance threshold will be set at 0.05 (two-sided), with 95% confidence intervals.

## Discussion

The results of this study will provide preliminary evidence regarding the efficacy and feasibility of Tai Chi as an additional rehabilitative intervention for stroke patients in the recovery phase. Furthermore, if the results are positive, this study will contribute to the establishment of further guidance in applying Tai Chi rehabilitation programs during the early stages of stroke.

The studies conducted by Taylor *et al*. and Au-Yeung *et al*. also applied Tai Chi rehabilitation programs for stroke patients, and their results indicated that Tai Chi was helpful[16–18]. However, in these previous studies, Tai Chi practice was geared mainly for the sequelae stage rather than the recovery phase of stroke. As recent studies have suggested, stroke rehabilitation should be started as soon as the patient’s vital signs become stable[34, 35]. Thus, it would be more reasonable and closer to clinical reality to apply Tai Chi for stroke rehabilitation in the early stages of stroke.

In most previous studies, researchers applied only scale evaluations to measure the outcomes of stroke rehabilitation. Few of them have introduced objective outcome measurements. In the present study, by using the footscan gait system, we will measure several objective parameters, including the plantar force distribution, impulse, maximum force, maximum pressure and time of walk phase, which will provide objective evaluation of the effects of different rehabilitation programs on lower-extremity function.

One limitation concerns the fact is that this is only a preliminary single-center study. Another limitation is that the therapist is not blinded in the present trial. A blinded study involving Tai Chi is challenging to conduct because it is almost impossible to blind the therapist and patients.

## Trial status

This trial is currently ongoing and 6 participants are still in need to complete the whole study.

## Authors’ original submitted files for images

Below are the links to the authors’ original submitted files for images. Authors’ original file for figure 1

## Abbreviations

BBS: Berg Balance Scale
BI: Barthel Index
FMA: Fugl-Meyer Assessment
NIHSS: National Institutes of Health Stroke Scale
SS-QOL: Stroke-Specific Quality of Life Scale.
