# Supplementary figures and images for: Applying Tai Chi as a rehabilitation program for stroke patients in the recovery phase: study protocol for a randomized controlled trial
Source: Trials. 2014 Dec 11;15:484. doi: 10.1186/1745-6215-15-484 (PMC4295286; doi:10.1186/1745-6215-15-484)

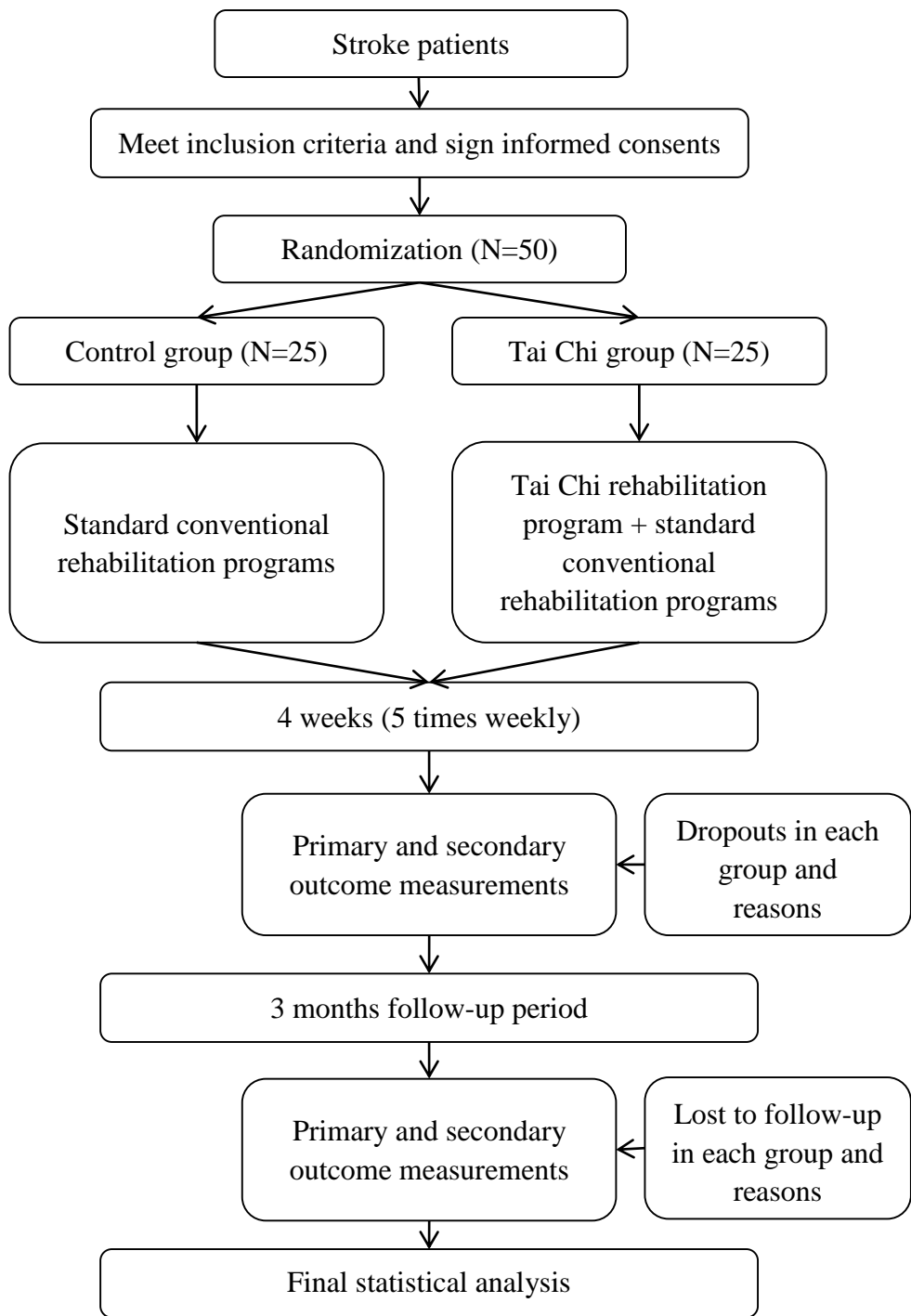

Supplement: Supplementary file 1 — Authors’ original file for figure 1 [file 13063_2014_2377_MOESM1_ESM.pdf]
